# Association between the Naples Prognostic Score and cognitive function in older adults: validation in Alzheimer’s disease and vascular dementia

**DOI:** 10.3389/fnut.2026.1761323

**Published:** 2026-02-23

**Authors:** Hao Yang, Sheng Zhang, Liping Zhong

**Affiliations:** 1 Department of Oncology, Huzhou Central Hospital, Fifth School of Clinical Medicine of Zhejiang Chinese Medical University, Huzhou, Zhejiang, China; 2 Department of Oncology, Huzhou Central Hospital, Affiliated Central Hospital of Huzhou University, Huzhou, Zhejiang, China; 3 Department of Neurology, Huzhou Central Hospital, Fifth School of Clinical Medicine of Zhejiang Chinese Medical University, Huzhou, Zhejiang, China; 4 Department of Neurology, Huzhou Central Hospital, Affiliated Central Hospital of Huzhou University, Huzhou, Zhejiang, China

**Keywords:** aged, cognitive impairment, dementia, Naples Prognostic Score (NPS), NHANES

## Abstract

**Background:**

Accumulating evidence indicates that systemic inflammation and metabolic dysregulation might contribute to cognitive impairment. The Naples Prognostic Score (NPS), a composite measure integrating albumin, total cholesterol (TC), neutrophil-to-lymphocyte ratio (NLR), and lymphocyte-to-monocyte ratio (LMR), provides a succinct profile of systemic inflammatory-metabolic status. This study investigated the association between the NPS and cognitive impairment in elderly Americans.

**Methods:**

We analyzed data from 2,595 participants (age ≥60) in the 2011–2014 National Health and Nutrition Examination Survey (NHANES). Cognitive function was evaluated using Consortium to Establish a Registry for Alzheimer’s Disease (CERAD), Animal Fluency Test (CFDAST), and Digit Symbol Substitution Test (CFDDS). Cognitive impairment was defined as scoring in the lowest quartile. Multivariate logistic regression, linear trend analysis, and subgroup analysis were used to assess the association between NPS and cognitive impairment. Furthermore, the distribution of NPS across different dementia subtypes was validated in an independent clinical cohort comprising 189 clinically diagnosed patients.

**Results:**

After full adjustment, higher NPS scores were associated with an increased risk of cognitive impairment. A significant association was observed specifically with executive function assessed by CFDAST (OR = 2.03, 95% CI: 1.18–3.51). Significant dose–response relationships were found for both CFDAST and CFDDS (*P* for trend = 0.007 and 0.025, respectively). In the clinical cohort, NPS levels were similarly elevated in patients with Alzheimer’s disease (AD) and vascular dementia (VaD) (mean 3.11 vs. 3.03, *p* = 0.432), suggesting that systemic inflammatory-metabolic dysregulation may constitute a common pathological mechanism across multiple subtypes of dementia.

**Conclusion:**

Higher NPS scores are independently associated with executive dysfunction in older adults. Although this scoring system has limited specificity in differentiating dementia subtypes, it shows potential as a screening tool for identifying populations at high risk of executive dysfunction. This underscores the potential of interventions targeting underlying inflammatory and metabolic pathways as a transdiagnostic strategy. Nevertheless, the predictive utility of the NPS requires comprehensive validation through prospective studies.

## 1 Introduction

Cognitive degenerative diseases, particularly Alzheimer’s disease and related dementias, have emerged as major global public health challenges. With accelerating population aging, the number of dementia patients worldwide is projected to rise from 57 million in 2019 to 152 million by 2050 (1). In the United States, approximately 11% of individuals aged 65 and older are diagnosed with Alzheimer’s disease (AD), and the prevalence rate among those over 85 reaches a striking 33% (2). These conditions not only cause severe cognitive impairment and reduced quality of life for patients, but also impose substantial economic burdens on families and society, with projected related healthcare expenditures in the United States reaching a staggering $345 billion in 2023.

However, early prediction and intervention for cognitive degenerative diseases remain significant challenges. First, commonly used diagnostic methods—such as cerebrospinal fluid testing and positron emission tomography (PET) scans—are invasive, expensive, or limited in availability, which restricts their utility in large-scale population screening (3). Second, existing predictive models predominantly rely on genetic factors (such as the APOE ε4 allele) or single biomarkers, which have limited predictive efficacy and clinical applicability (4). Notably, approximately 40% of dementia cases are linked to modifiable risk factors—including vascular risk factors, lifestyle, and air pollution (5)—posing challenges for developing early screening prediction systems for cognitive impairment.

These unmet clinical needs underscore the imperative to develop novel predictive tools. Ideal predictors should possess three key characteristics: (1) reliance on routinely available biomarkers; (2) integration of multiple pathophysiological pathways (e.g., inflammation, metabolic abnormalities); and (3) suitability for large-scale population screening. Therefore, exploring composite scoring systems (e.g., NPS) that combine biomarkers of inflammation and metabolism (e.g., albumin, cholesterol) may provide innovative solutions for early detection of cognitive degenerative diseases.

Recent studies have increasingly revealed the pivotal roles of systemic inflammation and metabolic status in the onset and progression of cognitive impairment. As a sensitive marker of systemic inflammatory response, the neutrophil-to-lymphocyte ratio (NLR) has been found to show significant correlation with cognitive decline in multiple studies (6). A cohort study involving 3,000 elderly individuals revealed that those with NLR > 2.5 had an 80% higher risk of developing mild disease (95% CI: 1.3–2.5) (7). Similarly, Li et al. reported that older Americans with elevated NLR values had a 5% higher risk of developing cognitive impairment (8). The lymphocyte-to-monocyte ratio (LMR), which reflects immune regulation, also shows clinical relevance: data from the U.S. National Institutes of Health indicate that a lower LMR (particularly ≤3.0) is linked to accelerated cognitive decline in patients with AD (9). With regard to metabolic biomarkers, low serum albumin (< 38 g/L) may promote amyloid-beta accumulation, thereby increasing dementia risk in AD (10). Additionally, low total cholesterol (< 160 mg/dL) has been associated with reduced gray matter volume in the medial temporal lobe, potentially impairing executive function (11). Collectively, these findings suggest that inflammatory and metabolic dysregulation may jointly contribute to neurodegeneration through mechanisms including blood–brain barrier disruption, amplified neuroinflammatory responses, and impaired neuronal repair. However, current research predominantly focuses on isolated biomarkers, failing to fully capture the synergistic effects of combined inflammatory-metabolic profiles.

The Naples Prognostic Score (NPS), initially proposed by Galizia et al. (12), was originally developed as a prognostic tool in colorectal cancer. In the context of aging and cognitive impairment, we propose to reinterpret this score as a composite biomarker of systemic immunometabolic status. Its components—serum albumin, TC, NLR, and lymphocyte-to-monocyte ratio (LMR)—are readily available in clinical practice. Critically, albumin and cholesterol levels are not merely nutritional markers but are heavily influenced by systemic inflammation (e.g., albumin as a negative acute-phase reactant) and metabolic homeostasis. Combined with the direct inflammatory signals from NLR and LMR, the NPS thus offers an integrated snapshot of the host’s inflammatory-metabolic milieu. This system boasts distinctive advantages for our research: (1) it utilizes routinely available clinical tests, ensuring broad accessibility; (2) it captures multiple interrelated pathophysiological pathways relevant to neurodegeneration; and (3) the cutoff values determined by the MaxStat algorithm (e.g., NLR ≥ 2.96) demonstrate optimal discriminative power. This study innovatively applies and reconceptualizes the NPS in the context of cognitive impairment, aiming to investigate its associations with specific cognitive domains (e.g., memory and executive function), establish its predictive utility, and explore whether this integrated systemic profile can inform personalized prevention and intervention strategies.

## 2 Methods

### 2.1 Data sources and population

The National Health and Nutrition Examination Survey (NHANES) is a population-based comprehensive study designed to assess the health and nutritional status of children and adults. NHANES health examinations are conducted by medical professionals at Mobile Examination Centers (MECs), focusing on collecting baseline physical examinations, biochemical tests, and other medical information. All data are publicly available through the official website.^1^ The study protocol has been approved by the National Center for Health Statistics (NCHS) Research Ethics Review Committee, and all participants have provided informed consent.

**Figure 1:**
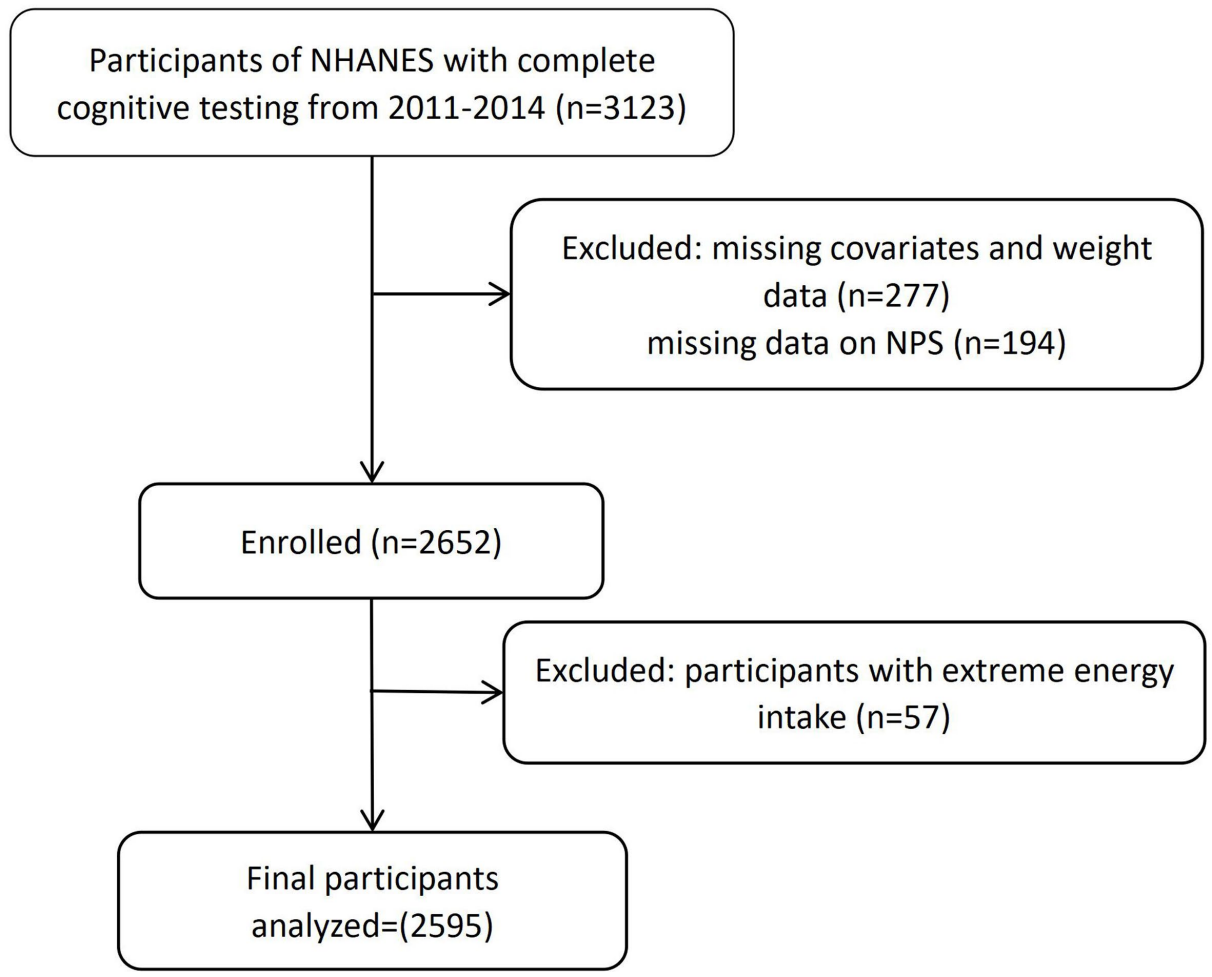
Flowchart for the selection of eligible participants.

This study analyzed NHANES data from 2011 to 2014. We excluded participants lacking NPS assessment data (serum albumin, TC, and complete blood count) (*n* = 194); those with extreme energy intake (men: >4,200 kcal/day or <800 kcal/day; women: > 3,500 kcal/day or < 500 kcal/day) (*n* = 57); and those with missing covariate information (*n* = 277). After these exclusions, a total of 2,595 participants were included in the final analysis (Figure 1). Ethical approval was granted by the NCHS Research Ethics Review Board (ERB), and all participants provided informed consent in accordance with ethical guidelines.

### 2.2 NPS score construction

The NPS score was calculated according to the method established by Galizia et al., based on four biomarkers: serum albumin, total cholesterol (TC), NLR, and lymphocyte-to-monocyte ratio (LMR). Only total cholesterol was available in the NHANES dataset for this analysis; lipid subfractions were not considered. The optimal cutoff values for NLR and LMR were determined using MaxStat analysis. Each component scored one point if it met the following abnormal criteria: serum albumin <40 g/L, TC ≤ 180 mg/dL, NLR ≥ 2.96, or LMR ≤ 4.44; otherwise, it scored zero points. The total NPS was the sum of points from all four parameters, ranging from 0 to 4. Based on prior studies, participants were categorized into three groups: Group 0 (score = 0), Group 1 (score = 1–2), and Group 2 (score = 3–4).

### 2.3 Cognitive function assessment

Cognitive function was assessed in participants aged 60 years and older using three standardized neuropsychological tests. The Consortium to Establish a Registry for Alzheimer’s Disease (CERAD) test evaluates immediate and delayed recall of verbal information through three consecutive learning trials and one delayed recall trial, with total scores ranging from 0 to 40. The Animal Fluency Test (CFDAST) measures semantic memory and verbal fluency by asking participants to name as many animals as possible within one minute; the score is the total number of correct responses (13). The Digit Symbol Substitution Test (CFDDS) assesses processing speed, visuomotor coordination, sustained attention, and working memory (14, 15). Participants were required to match symbols to numbers using a reference key within 120 s, and the total score was determined by the number of correctly completed items, up to a maximum of 133 points. Higher scores on all tests indicate better cognitive performance.

In the absence of universally accepted diagnostic criteria for these tests, we operationalized cognitive impairment as a score in the lowest quartile of our sample distribution for each test (16). It should be noted that this constitutes a relative, sample-specific threshold for poorer cognitive performance, not a clinical cutoff for diagnosing dementia or cognitive impairment.

### 2.4 Covariates

Baseline covariates included demographics, lifestyle factors, and clinical conditions. Demographic variables comprised age (years), gender (male/female), education level (<high school, high school, >high school), race/ethnicity (Mexican American, other Hispanic, non-Hispanic White, non-Hispanic Black, other), and body mass index (BMI; <25.0, 25.0–29.9, ≥30.0 kg/m^2^). Smoking status was categorized as never (<100 cigarettes in lifetime), former (≥100 cigarettes but quit), or current (≥100 cigarettes and currently smoking). Alcohol consumption was classified as non-drinker, low-to-moderate (men <28 g/day, women <14 g/day), or heavy (men ≥28 g/day, women ≥14 g/day). Daily intake of caffeine, energy, total fat, and total sugar was averaged from two 24 h dietary recall interviews. Hypertension was defined as systolic blood pressure ≥140 mmHg, diastolic blood pressure ≥90 mmHg, self-reported diagnosis, or use of antihypertensive medication. Diabetes was defined as self-reported diagnosis, use of insulin or oral hypoglycemic agents, HbA1c ≥ 6.5%, or fasting plasma glucose ≥126 mg/dL.

### 2.5 Statistical analysis

All analyses were performed in R (version 4.4.1). Considering the complex sampling design of NHANES, all analyses applied survey weights to ensure the national representativeness of the results. For the combined 2011–2014 cycles, the original 2-year examination weights (WTMEC2YR) were halved to create adjusted weights.

Continuous variables are reported as weighted mean (standard error, SE), and categorical variables as unweighted count (weighted percentage). Between-group differences were assessed using analysis of variance (ANOVA) for continuous variables and the Rao–Scott adjusted chi-square test for categorical variables.

Cognitive outcomes were assessed using three standardized tests: CERAD, CFDAST, and CFDDS. Given the exploratory nature of this first investigation of NPS in cognitive aging, we considered all three cognitive domains as equally important exploratory outcomes. Cognitive impairment was defined as scoring in the lowest quartile of each test, creating a binary outcome for logistic regression. To address multiple comparisons, we report unadjusted *p* values and indicate results that remain significant after a conservative Bonferroni correction for three tests (adjusted *α* = 0.0167).

A multivariate weighted logistic regression model was used to examine the association between NPS and cognitive function, constructing three progressively adjusted models: a crude model without covariate adjustments; Model 1 adjusted for age, gender, and race/ethnicity; Model 2 further adjusted for education level, smoking status, alcohol consumption, caffeine intake, BMI category, hypertension, diabetes, and energy intake. Results are presented as odds ratios (OR) with 95% confidence interval (CI). Trend analysis was performed by modeling NPS as a continuous variable.

To evaluate potential effect modification, interaction terms between NPS and each stratification variable (gender and age groups [60–75 vs. >75 years]) were included in the models; corresponding *p* values are reported.

Sensitivity analyses were conducted to assess robustness: (1) treating cognitive test scores as continuous outcomes in survey-weighted linear regression models, and (2) redefining cognitive impairment using a stricter cutoff (lowest decile) in logistic regression models.

A two-sided *p* value < 0.05 defined statistical significance. Analyses employed the following packages: survey, tidyverse, broom, and gt.

### 2.6 Clinical validation cohort

To validate findings from the NHANES population, patients from the Department of Neurology at Huzhou Central Hospital were included. This included 87 patients with clinically diagnosed Alzheimer’s dementia (AD) and 102 patients with vascular dementia (VaD). Patients with severe hepatic/renal dysfunction, critical infections, or other major neurological disorders were excluded. Fasting blood samples were obtained to calculate the NPS. This study was approved by the Ethics Committee of Huzhou Central Hospital, and the requirement for informed consent was waived. The Mann–Whitney U test was used to compare NPS values between the two groups, and the common language effect size (CLES) was calculated to assess the magnitude of the effect. Data visualization was performed using the ggplot2 package in R to generate boxplots illustrating the distribution of NPS scores in both groups.

## 3 Results

### 3.1 Characteristics of study participants

This study included a final sample of 2,595 participants from the NHANES 2011–2014 dataset. Table 1 compares baseline characteristics by NPS group, with categorical variables presented as weighted percentages and continuous variables as weighted means (standard errors).

**Table 1:** Baseline characteristics by NPS group^a^.

Variable	0	1–2	3–4	*p* value
	*n* = 341	*n* = 1,776	*n* = 478	
Age, years	67.45 (0.4)	68.89 (0.22)	71.44 (0.4)	<0.001
Gender				<0.001
Male	92 (20.9%)	901 (47.7%)	280 (56.9%)	
Female	249 (79.1%)	875 (52.3%)	198 (43.1%)	
Race				0.005
Mexican American	36 (4.8%)	157 (3.2%)	41 (3.3%)	
Other Hispanic	48 (5.7%)	182 (3.5%)	37 (2.8%)	
Non-Hispanic White	118 (71.4%)	898 (82.0%)	266 (81.0%)	
Non-Hispanic Black	91 (9.9%)	396 (7.4%)	98 (7.6%)	
Other race	48 (8.2%)	143 (4.0%)	36 (5.3%)	
Education level				0.418
<High school	36 (5.4%)	210 (5.5%)	57 (6.5%)	
Complete high school	48 (9.7%)	239 (9.8%)	73 (12.5%)	
>High school	257 (85.0%)	1,327 (84.7%)	348 (81.0%)	
Smoking status				< 0.001
Never smoker	203 (60.9%)	866 (49.9%)	208 (40.2%)	
Former smoker	93 (26.4%)	679 (39.7%)	217 (47.8%)	
Current smoker	45 (12.7%)	231 (10.3%)	53 (12.0%)	
Alcohol consumption				0.659
Heavy	31 (12.8%)	206 (15.2%)	44 (13.7%)	
Low-to-moderate	45 (17.8%)	257 (16.3%)	62 (13.1%)	
Nondrinker	265 (69.3%)	1,313 (68.5%)	372 (73.2%)	
Caffeine intake (mg/day)				0.588
<400	305 (89.0%)	1,546 (86.5%)	423 (89.4%)	
≥400	17 (7.7%)	116 (9.1%)	26 (7.1%)	
None	19 (3.3%)	114 (4.4%)	29 (3.6%)	
BMI category (kg/m^2^)				0.025
<25.0	107 (37.3%)	465 (24.9%)	132 (27.1%)	
25.0–29.9	121 (36%)	636 (36.4%)	145 (32%)	
>29.9	113 (26.7%)	675 (38.7%)	201 (40.9%)	
Hypertension				0.002
Yes	219 (55%)	1,250 (66.1%)	373 (75.5%)	
No	122 (45%)	526 (33.9%)	105 (24.5%)	
Diabetes				<0.001
Yes	82 (16.4%)	495 (22.2%)	208 (37%)	
No	259 (83.6%)	1,281 (77.8%)	270 (63%)	
Sugar intake				0.148
Low	6 (0.6%)	53 (2%)	18 (3.1%)	
Medium	42 (10.9%)	238 (13.1%)	76 (16.4%)	
High	293 (88.5%)	1,485 (84.9%)	384 (80.5%)	
Fat intake				0.509
Low	133 (30%)	523 (23.5%)	134 (24.4%)	
Medium	128 (39.3%)	675 (40.2%)	182 (39.2%)	
High	80 (30.8%)	578 (36.4%)	162 (36.4%)	
Energy intake (kcal/day)				0.127
500–1,578	165 (43.1%)	678 (32.2%)	189 (36.1%)	
1,579–2,259	113 (34.0%)	694 (42.4%)	182 (40.0%)	
2,260–4,200	63 (22.9%)	404 (25.4%)	107 (23.9%)	
NLR	1.58 (0.06)	2.31 (0.04)	3.96 (0.12)	<0.001
LMR	5.85 (0.08)	3.52 (0.04)	2.48 (0.06)	<0.001
CERAD	27.11 (0.46)	26.26 (0.4)	23.79 (0.29)	<0.001
CFDAST	19.13 (0.51)	18.57 (0.21)	15.83 (0.33)	<0.001
CFDDS	55.9 (1.4)	53.99 (0.65)	46.26 (0.99)	<0.001

a Continuous variables are presented as weighted mean (SE); categorical variables are presented as count (weighted %). All analyses accounted for the complex survey design of NHANES using appropriate sample weights. *P* values were derived from survey-weighted linear regression for continuous variables and survey-weighted chi-square tests for categorical variables. NPS, Naples Prognostic Score; NLR, neutrophil-to-lymphocyte ratio; LMR, lymphocyte-to-monocyte ratio; CERAD, consortium to establish a registry for Alzheimer’s disease; CFDAST, animal fluency test; CFDDS, digit symbol substitution test; BMI, body mass index.

Among the participants, 341 (13.1%) scored NPS 0, 1,776 (68.4%) scored NPS 1–2, and 478 (18.4%) scored NPS 3–4. Compared with the NPS 0 group, participants in the NPS 3–4 group were older (71.44 vs. 67.45 years), had a higher proportion of males (56.9% vs. 20.9%), and included a greater representation of Non-Hispanic White participants (81.0% vs. 71.4%). This group also exhibited a significantly higher prevalence of hypertension (75.5% vs. 55.0%) and diabetes (37.0% vs. 16.4%). Inflammatory biomarkers showed notable differences: the NLR was significantly higher (3.96 vs. 1.58), while the lymphocyte-to-monocyte ratio was markedly lower (2.48 vs. 5.85). Regarding cognitive function, the NPS 3–4 group had significantly lower scores on the CERAD (23.79 vs. 27.11), the CFDAST (15.83 vs. 19.13), and the CFDDS (46.26 vs. 55.90), with all differences statistically significant (*p* < 0.001).

### 3.2 Multivariate regression analysis of NPS and cognitive function

Table 2 illustrates the relationship between NPS groupings and cognitive dysfunction. After comprehensive adjustment for potential confounders—including age, gender, race, education level, smoking status, alcohol consumption, caffeine intake, BMI category, hypertension, diabetes, and energy intake—a domain-specific association was observed: higher NPS scores (3–4 points) were significantly associated with cognitive dysfunction assessed by the CFDAST (executive function/language fluency: OR 2.03, 95% CI 1.18–3.51, *p* = 0.016). This association remained statistically significant even after applying a conservative Bonferroni correction for three tests (adjusted *α* = 0.0167). In contrast, no significant associations were found between NPS groupings and cognitive dysfunction assessed by the CERAD test (verbal memory: OR 1.43, 95% CI 0.76–2.69, *p* = 0.230) or the CFDDS test (processing speed: OR 1.78, 95% CI 0.95–3.34, *p* = 0.069).

**Table 2:** Association between NPS group and cognitive impairment^a^.

NPS group	Crude model		Model 1		Model 2	
	OR (95% CI)	*P* value	OR (95% CI)	*P* value	OR (95% CI)	*P* value
CERAD						
0	Reference		Reference		Reference	
1–2	1.47 (0.98–2.22)	0.061	1.26 (0.84–1.89)	0.251	1.27 (0.80–2.01)	0.277
3–4	2.28 (1.34–3.86)	**0.003**	1.44 (0.81–2.56)	0.208	1.43 (0.76–2.69)	0.230
*P* for trend		**0.002**		0.230		0.247
CFDAST						
0	Reference		Reference		Reference	
1–2	1.06 (0.74–1.53)	0.731	1.13 (0.76–1.67)	0.536	1.11 (0.72–1.72)	0.597
3–4	2.41 (1.46–3.96)	**0.001**	2.19 (1.32–3.64)	**0.004**	2.03 (1.18–3.51)	**0.016**
*P* for trend		**<0.001**		**0.001**		**0.007**
CFDDS						
0	Reference		Reference		Reference	
1–2	0.97 (0.62–1.53)	0.896	0.94 (0.55–1.60)	0.820	0.86 (0.45–1.65)	0.617
3–4	2.31 (1.35–3.95)	**0.003**	1.93 (1.10–3.38)	**0.024**	1.74 (0.94–3.24)	0.069
*P* for trend		**<0.001**		**0.007**		**0.025**

a Cognitive impairment was defined as scoring in the lowest quartile of each cognitive test. Crude model: no covariates adjusted. Model 1: adjusted for age, gender, and race. Model 2: additionally adjusted for education level, smoking status, alcohol consumption, caffeine intake, BMI category, hypertension, diabetes, and energy intake. Bold values indicate statistically significant associations (*P* < 0.05). NPS, Naples Prognostic Score; OR, odds ratio; CI, confidence interval; CERAD, total score from consortium to establish a registry for Alzheimer’s disease test; CFDAST, animal fluency test score; CFDDS, digit symbol substitution test score.

Trend tests indicated significant dose–response relationships for CFDAST (*P* for trend = 0.007) and CFDDS (*P* for trend = 0.025, though borderline) after full adjustment, suggesting a graded increase in risk with higher NPS scores. In contrast, no such trend was observed for CERAD (*P* for trend = 0.247). For CFDAST, the highest NPS group (3–4 points) exhibited significantly elevated cognitive impairment risk across all models (Crude Model: OR 2.41, 95% CI 1.46–3.96, *p* = 0.001; Model 1: OR 2.19, 95% CI 1.32–3.64, *p* = 0.004; Model 2: OR 2.03, 95% CI 1.18–3.51, *p* = 0.016). Notably, the dose–response trend for CFDAST also remained significant after Bonferroni correction (*p* = 0.007 < 0.0167). Similarly, for CFDDS, the highest NPS group showed significantly increased odds in the crude model (OR 2.31, 95% CI 1.35–3.95, *p* = 0.003) and Model 1 (OR 1.93, 95% CI 1.10–3.38, *p* = 0.024), although the association became marginally non-significant in the fully adjusted model (OR 1.78, 95% CI 0.95–3.34, *p* = 0.069).

**Figure 2:**
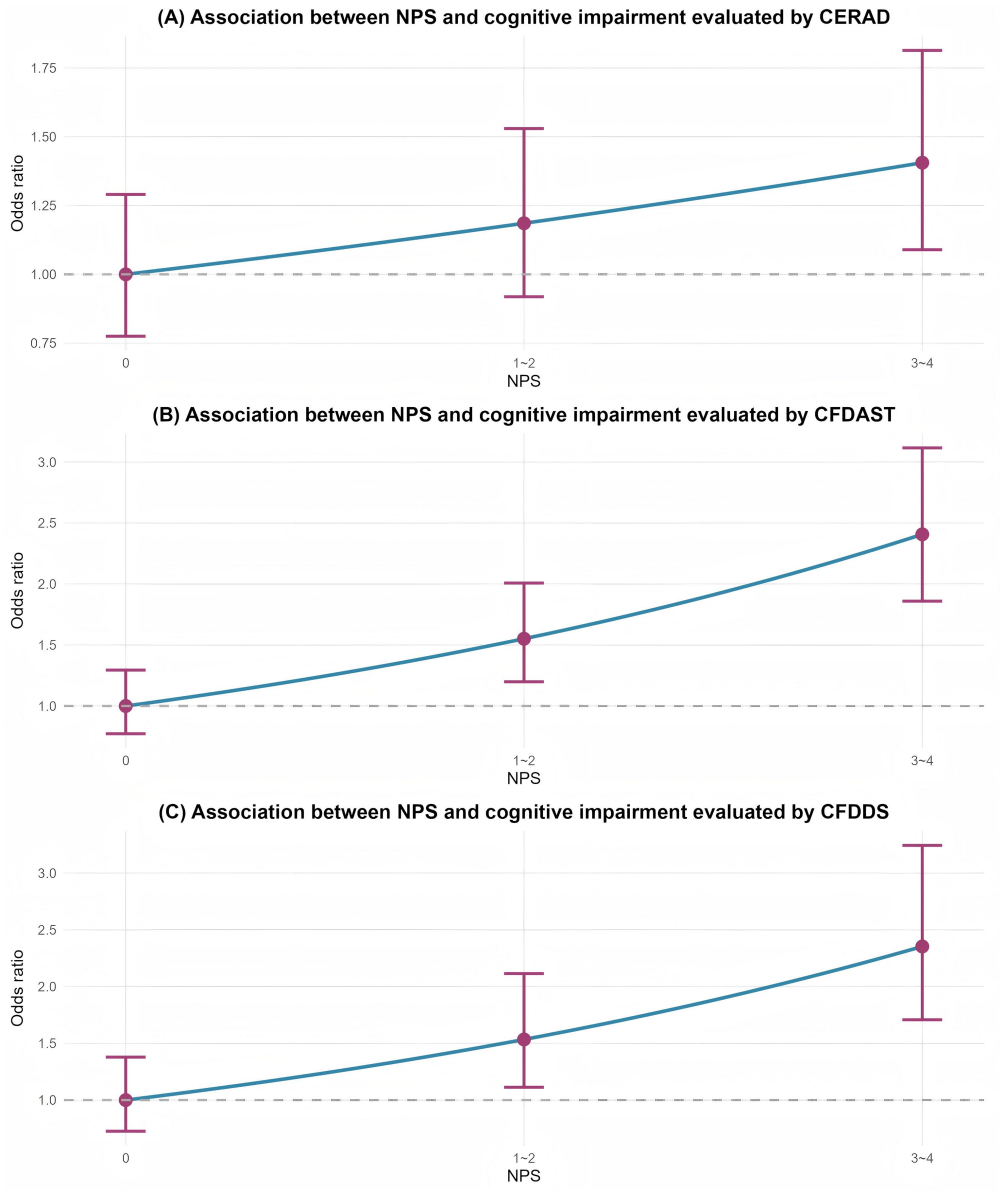
Dose–response relationship between NPS scores and cognitive impairment risk in three cognitive tests: **(A)** Cognitive performance assessed by the CERAD test; **(B)** Cognitive performance assessed by the CFDAST test; **(C)** Cognitive performance assessed by the CFDDS test. Linear trend analysis revealed significant associations between higher NPS scores and CFDAST (*P* for trend = 0.007) and CFDDS (*P* for trend = 0.025) after full adjustment for confounding factors (Model 2). No significant association was observed for CERAD (*P* for trend = 0.247). These findings indicate that cognitive impairment risk increases progressively with higher NPS scores, particularly in tests assessing language fluency and processing speed.

The dose–response relationship between NPS as a continuous variable and cognitive impairment risk across all three tests is visually presented in Figure 2.

These results indicate that higher NPS scores are associated with an increased risk of executive function-related cognitive impairment, even after rigorous adjustment for multiple confounding variables. The domain-specific pattern suggests that NPS may be more strongly related to frontal-executive circuits than to memory or processing speed networks.

### 3.3 Incremental predictive value of NPS

To validate the rationale for using the composite NPS over individual biomarkers, we compared the model fit (AIC) and discrimination (AUC) of the NPS risk stratification system (Groups 0, 1–2, 3–4) against models based on continuous individual components (NLR, LMR, albumin, and TC) for executive function (CFDAST). As shown in Supplementary Table S1, the NPS Group model demonstrated the superior predictive performance among all tested models, achieving the lowest AIC (1,970.55) and the highest AUC (0.7323). Notably, the composite NPS model exhibited a significant improvement in model fit (ΔAIC >10) compared to the second-best model (TC, AIC = 1,980.99) and the inflammatory marker NLR alone (AIC = 1,983.58). These results suggest that the NPS classification effectively integrates inflammatory and metabolic risks, providing a more robust screening tool than relying on any single continuous biomarker.

### 3.4 Sensitivity analyses

To assess the robustness of our primary findings, we conducted two sensitivity analyses. First, when cognitive test scores were analyzed as continuous outcomes in fully adjusted linear regression models, each 1-point increase in NPS (range 0–4) was associated with significantly lower scores on all three cognitive tests: CERAD (*β* = −0.42, 95% CI: −0.83 to −0.02, *p* = 0.042), CFDAST (*β* = −0.75, 95% CI: −0.99 to −0.51, *p* < 0.001), and CFDDS (*β* = −1.29, 95% CI: −1.93 to −0.65, *p* = 0.001) (Supplementary Table S2). Second, when applying a more stringent cutoff (lowest decile, 10%) to define cognitive impairment, the direction and magnitude of associations were similar to the primary analysis (Supplementary Table S3). For CFDAST, the odds ratio for the highest NPS group (3–4) versus the lowest (0) was 2.11 (95% CI: 0.85–5.18, *p* = 0.096) in the fully adjusted model, compared to 2.03 (1.18–3.51, *p* = 0.016) in the primary analysis, with the dose–response trend remaining significant (*P* for trend = 0.046). These sensitivity analyses confirm that the inverse association between higher NPS scores and poorer cognitive performance is robust across different analytical approaches.

### 3.5 Exploratory stratified analysis

To explore potential variations in different population subgroups, we conducted stratified analyses by age and gender (Supplementary Table S4). Although point estimates varied slightly across subgroups, formal interaction tests did not reach statistical significance (all p for interaction >0.05). This suggests that the association between NPS and executive function impairment is generally consistent across age groups and genders, and no definitive effect modification was observed in this population.

### 3.6 Exploratory clinical validation analysis

In the expanded exploratory clinical validation cohort (*n* = 189), NPS scores were highly similar between the AD group and the VaD group. The mean NPS score was 3.11 ± 1.00 in the AD group and 3.03 ± 0.97 in the VaD group, with both groups exhibiting a median of 3. The Mann–Whitney U test revealed no statistically significant difference between the two groups (*p* = 0.432). The common language effect size (CLES) was 0.469, indicating that the probability of a randomly selected individual from the AD group having a higher NPS score than one from the VaD group was 46.9% (Table 3).

**Table 3:** Comparison of NPS between AD and VaD groups in the exploratory clinical cohort^a^.

Group	*n*	Mean ± SD	Median	Mann–Whitney U	*P* value	Effect size (CLES)
AD	87	3.11 ± 1.00	3	4,159	0.432	0.469
VaD	102	3.03 ± 0.97	3	–	–	

a Continuous variables are presented as mean ± standard deviation. P value was calculated via Mann–Whitney U test. CLES (Common Language Effect Size) indicates the probability that a randomly selected score from one group is higher than a score from the other. NPS, Naples Prognostic Score; AD, Alzheimer’s disease; VaD, vascular dementia.

**Figure 3:**
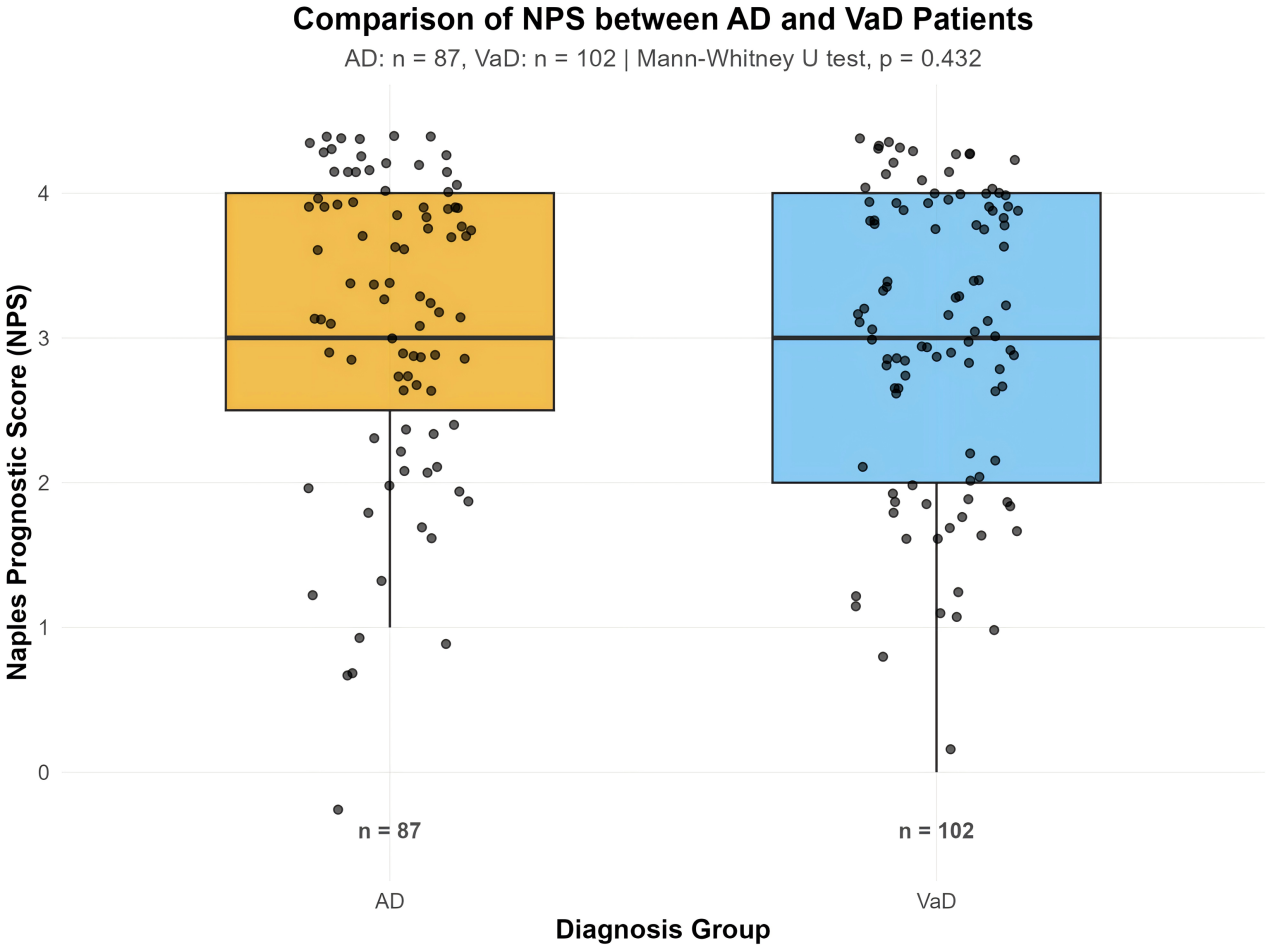
Boxplots display the median and distribution of NPS for AD (*n* = 87) and VaD (*n* = 102) patients, with individual scores shown as black dots. No significant difference was observed between the two groups (Mann–Whitney *U* test, *p* > 0.05).

The distribution of NPS scores in the two groups was visually presented through box plots (Figure 3). As shown in the figure, the data distribution ranges highly overlapped, further confirming the statistical result that there was no significant difference in NPS between the two groups. These findings suggest a shared inflammatory-metabolic burden across these dementia subtypes.

## 4 Discussion

This study demonstrates that a higher Naples Prognostic Score (NPS)—a composite marker of systemic inflammatory-metabolic dysregulation—is associated with an increased risk of domain-specific cognitive decline in older adults, with the most robust association observed for executive function. To our knowledge, this is the first large-scale study to establish this link. The association remained significant after rigorous adjustment and correction for multiple comparisons, and the composite NPS model outperformed models based on any of its individual components, validating its integrative value. Exploratory analysis in a clinical cohort revealed similarly elevated NPS in both AD and VaD patients, suggesting it captures a transdiagnostic risk profile.

A key conceptual advancement of this work is the reinterpretation of the NPS—beyond its original oncological context—as an indicator of a “systemic inflammatory-metabolic phenotype” relevant to cognitive aging. This framework is critical, as components like albumin are not mere nutritional markers but acute-phase reactants influenced by inflammation. The score’s strength lies in synthesizing information on inflammation (via NLR/LMR), metabolic status (cholesterol), and a hepatic carrier protein reflective of both processes (albumin), offering a more holistic risk assessment than any single biomarker. Importantly, this theoretical advantage was confirmed by our model comparisons: the composite NPS model demonstrated statistical superiority (lowest AIC, highest AUC) over models based on any of its individual components, validating its utility as an integrated risk indicator.

**Figure 4:**
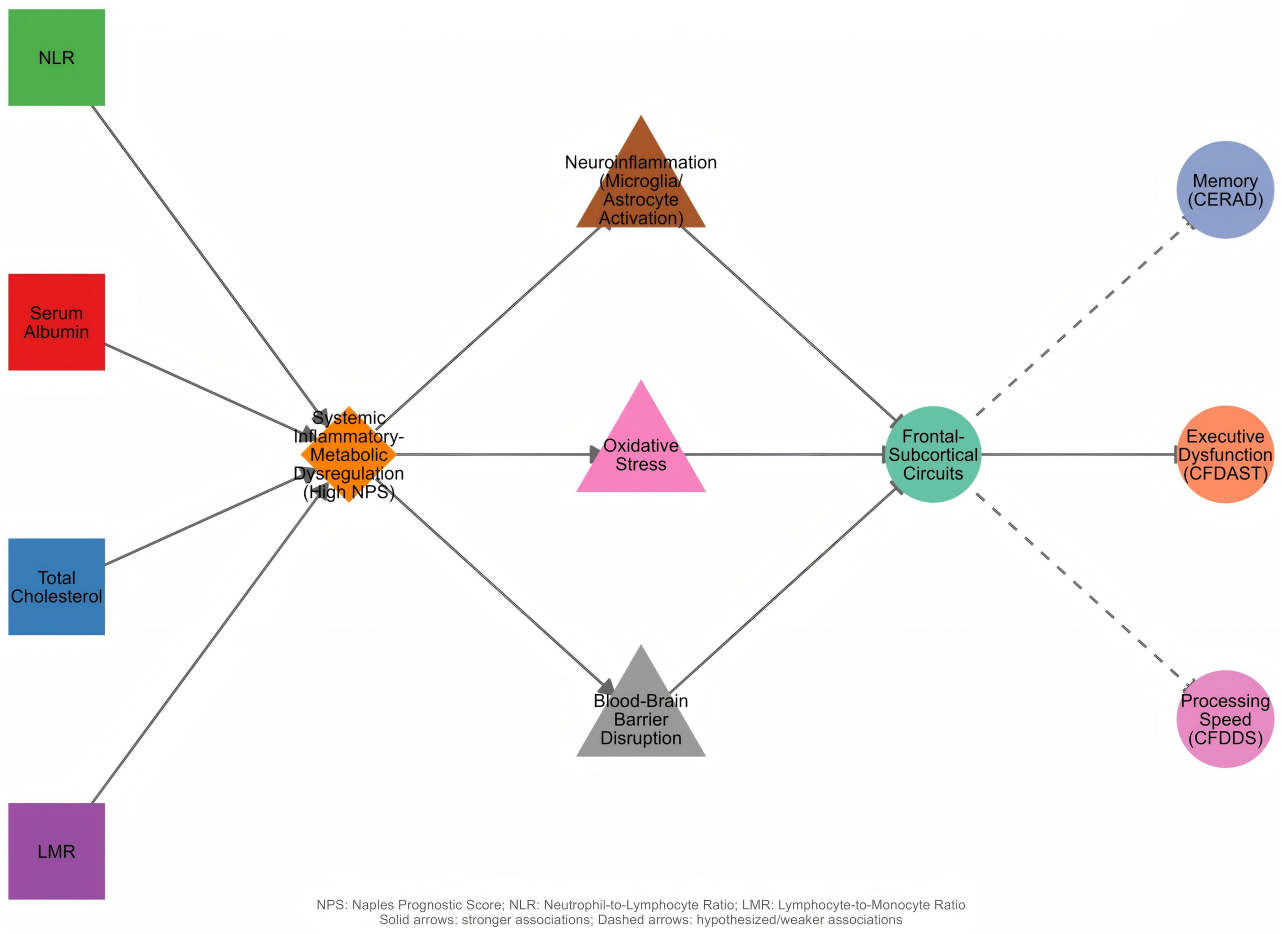
Proposed mechanistic framework linking Naples Prognostic Score to domain-specific cognitive dysfunction.

The underlying mechanisms linking NPS with cognitive function may involve multiple pathophysiological pathways (Figure 4). Specifically, serum albumin possesses a high binding capacity for amyloid beta (Aβ), facilitating its efflux from the central nervous system across the blood–brain barrier into the peripheral circulation. This mechanism helps maintain the dynamic equilibrium of Aβ between the brain and bloodstream, preventing protein misfolding and aggregation. Hypoalbuminemia compromises this “peripheral sink” clearance mechanism, leading to cerebral Aβ retention, which promotes amyloid plaque deposition and neurotoxicity. Concurrently, albumin exerts intrinsic antioxidant and anti-inflammatory effects; its depletion exacerbates oxidative stress and neuroinflammation, creating a synergistic interplay with Aβ pathology that collectively accelerates cognitive decline (17–19). Evidence from Tan et al. indicates that systemic inflammation, as reflected by elevated NLR and LMR, triggers the release of pro-inflammatory cytokines such as IL-6, TNF-*α*, and IL-1β, which facilitate the infiltration of peripheral immune cells into the central nervous system (CNS). Upon entering the CNS, these peripheral signals activate the NF-κB pathway via pattern recognition receptors (e.g., Toll-like receptors), driving the activation of microglia and astrocytes to release further neuroinflammatory mediators that directly compromise frontal lobe neurons. Mechanistically, elevated TNF-α downregulates the expression of the glutamate transporter EAAT2 on astrocytes, leading to glutamate accumulation in the synaptic cleft. This results in the over-activation of NMDA receptors, causing neuronal excitotoxicity and synaptic dysfunction. Furthermore, this chronic inflammatory milieu impairs Long-Term Potentiation (LTP) mechanisms, disrupting the synaptic plasticity and connectivity within the frontal cortex—the neural substrate essential for executive function. Collectively, these cascades precipitate frontal neuronal dysfunction, clinically manifesting as the decline in executive function observed in our high-NPS group (20). Collectively, these factors contribute to neurodegeneration through mechanisms including blood–brain barrier disruption, oxidative stress, and impaired neuronal repair. By summarizing these multifaceted risks into a single stratum, the NPS effectively flags a “high-inflammation, poor-metabolic” phenotype that might be missed by examining single markers in isolation.

The association between NPS and cognitive function demonstrated a clear domain-specific pattern. The significant association was observed specifically for executive function (CFDAST), which remained robust even after Bonferroni correction, but not for memory or processing speed. This differential sensitivity likely reflects the distinct neural substrates of these cognitive domains. While memory processes predominantly depend on the temporohippocampal region, executive functions rely heavily on frontal-subcortical circuits, particularly the dorsolateral prefrontal cortex (21, 22). Our findings suggest that these prefrontal networks may exhibit heightened vulnerability to the systemic inflammatory-metabolic dysregulation captured by the NPS.

Our exploratory clinical validation in an expanded cohort (*n* = 189) found no significant difference in NPS between AD and VaD groups. While these findings should be interpreted as hypothesis-generating, the lack of specificity reinforces the concept that the adverse systemic phenotype captured by NPS represents a shared pathological pathway or comorbidity burden across major dementia subtypes. This transdiagnostic nature positions NPS as a potential screener for a high-risk systemic state common in neurodegeneration, rather than a disease-specific biomarker.

This study benefits from several key strengths: a large, nationally representative sample, rigorous adjustment for a comprehensive set of confounders, and the demonstration of a clear dose–response relationship with a critical risk threshold. Furthermore, we provided a dual-layer validation of the NPS utility: statistically validating its incremental predictive value over individual components, and exploratorily verifying its relevance in an independent clinical cohort.

However, several important limitations must be acknowledged. First and foremost, the cross-sectional design precludes definitive conclusions about causal relationships between the systemic inflammatory-metabolic status and cognitive impairment, as reverse causation cannot be excluded. Specifically, cognitive dysfunction—particularly in executive domains—may undermine an individual’s capacity for healthful decision-making, chronic disease management, and treatment adherence, thereby exacerbating this adverse inflammatory-metabolic state. Second, our operational definition of cognitive impairment was based on the lowest quartile of test scores within the NHANES population. While this is a practical epidemiological approach, it serves as a proxy for poor performance rather than a clinical diagnosis. The observed odds ratios (e.g., ~2.0 for CFDAST) should be interpreted as reflecting an increased risk of being among the poorest performers on these specific tests. Nevertheless, our sensitivity analyses using continuous scores and a stricter cutoff (lowest decile) yielded consistent findings, supporting the robustness of the association. Third, regarding the generalizability of the scoring system, the NPS cut-off values applied in this analysis were primarily derived from Western populations (NHANES and original European studies). Given that physiological baselines for inflammatory and metabolic markers may vary across ethnic groups, the optimal thresholds for Asian populations need to be rigorously verified. Although our exploratory clinical cohort provides preliminary validation in a Chinese population, large-scale studies are required to establish ethnicity-specific scoring standards. Finally, the predictive value of the NPS for the longitudinal progression of cognitive impairment requires comprehensive validation through multi-center prospective studies.

## 5 Conclusion

Despite the aforementioned limitations, this study provides a comprehensive examination of the association between NPS and cognitive function among older adults in the United States, integrating population-based analysis with clinical validation. The findings demonstrate that elevated NPS scores are independently associated with an increased risk of executive function decline in this population. Our analysis confirms that the composite score offers superior predictive value compared to individual inflammatory or metabolic markers alone. Although the scoring system has limited specificity in differentiating dementia subtypes, it shows promise as a screening tool to identify older adults at higher risk of cognitive decline, based on a profile of systemic inflammation and metabolic dysregulation. Furthermore, it may facilitate the identification of transdiagnostic patient subgroups who are likely to benefit from interventions targeting these underlying inflammatory and metabolic pathways. The predictive utility of NPS warrants further validation through large-scale, prospective studies.

## Data Availability

The original contributions presented in the study are included in the article/Supplementary material, further inquiries can be directed to the corresponding author.

## Footnotes

1
https://www.cdc.gov/nchs/nhanes
