# Supplementary material for: Association between the Naples Prognostic Score and cognitive function in older adults: validation in Alzheimer’s disease and vascular dementia
Source: Front Nutr. 2026 Feb 23;13:1761323. doi: 10.3389/fnut.2026.1761323 (PMC12967939; doi:10.3389/fnut.2026.1761323)
Supplement: Supplementary file 1 [file Table_1.DOCX]

| Supplementary Table S1. Comparison of Model Fit and Discrimination between NPS Group and Individual Components for Executive Function Impairment. | | |
| --- | --- | --- |
| Model | AIC (Model Fit) | AUC (Discrimination) |
| **NPS Group (0, 1**–**2, 3**–**4)** | **1,970.55** | **0.7323** |
| Total Cholesterol | 1,980.99 | 0.7281 |
| Albumin | 1,981.30 | 0.7293 |
| NLR | 1,983.58 | 0.7265 |
| LMR | 1,986.72 | 0.7255 |
| All models were adjusted for age, gender, race, education level, smoking status, alcohol consumption, caffeine intake, BMI category, hypertension, diabetes, and energy intake. AIC was calculated based on model deviance, where lower values indicate better model fit. AUC indicates discriminative ability for executive function impairment (assessed by CFDAST). AIC: Akaike Information Criterion; AUC: Area Under the Curve; NPS: Naples Prognostic Score; NLR: Neutrophil-to-Lymphocyte Ratio; LMR: Lymphocyte-to-Monocyte Ratio. | | |

| Supplementary Table S2. Association between NPS (as a continuous variable) and Cognitive Test Scores in Fully Adjusted Linear Regression Models^1^ | | |
| --- | --- | --- |
| Cognitive Test | β (95% CI) | *P* value |
| CERAD | -0.42 (-0.83 to -0.02) | 0.042 |
| CFDAST | -0.75 (-0.99 to -0.51) | < 0.001 |
| CFDDS | -1.29 (-1.93 to -0.65) | 0.001 |
| ^1^ NPS was analyzed as a continuous variable ranging from 0 to 4. All models were fully adjusted for age, gender, race, education level, smoking status, alcohol consumption, caffeine intake, BMI category, hypertension, diabetes, and energy intake. β represents the change in cognitive test score associated with a 1-point increase in NPS. NPS: Naples Prognostic Score; CI: Confidence Interval; CERAD: total score from Consortium to Establish a Registry for Alzheimer's Disease test; CFDAST: Animal Fluency Test score; CFDDS: Digit Symbol Substitution Test score. | | |

| Supplementary Table S3. Association between NPS Group and Cognitive Impairment Defined by the Lowest Decile^1^ | | | | | | |
| --- | --- | --- | --- | --- | --- | --- |
| NPS Group | Crude Model | | Model 1 | | Model 2 | |
|  | OR (95% CI) | *P* value | OR (95% CI) | *P* value | OR (95% CI) | *P* value |
| CERAD | | | | | | |
| 0 | Reference |  | Reference |  | Reference |  |
| 1–2 | 1.00 (0.55–1.81) | 1.000 | 0.82 (0.46–1.47) | 0.490 | 0.82 (0.42–1.61) | 0.532 |
| 3–4 | 1.83 (0.96–3.46) | 0.063 | 1.09 (0.52–2.28) | 0.810 | 1.13 (0.47–2.70) | 0.765 |
| *P* for trend |  | 0.021 |  | 0.523 |  | 0.502 |
| CFDAST | | | | | | |
| 0 | Reference |  | Reference |  | Reference |  |
| 1–2 | 0.95 (0.54–1.67) | 0.844 | 0.97 (0.52–1.81) | 0.923 | 0.98 (0.52–1.85) | 0.951 |
| 3–4 | 2.63 (1.23–5.59) | 0.014 | 2.16 (0.93–5.02) | 0.072 | 2.11 (0.85–5.18) | 0.096 |
| *P* for trend |  | 0.003 |  | 0.025 |  | 0.046 |
| CFDDS | | | | | | |
| 0 | Reference |  | Reference |  | Reference |  |
| 1–2 | 1.12 (0.62–2.04) | 0.689 | 1.25 (0.65–2.40) | 0.479 | 1.15 (0.57–2.34) | 0.669 |
| 3–4 | 2.18 (1.18–4.02) | 0.014 | 1.98 (0.95–4.09) | 0.065 | 1.89 (0.81–4.38) | 0.123 |
| *P* for trend |  | 0.002 |  | 0.027 |  | 0.065 |
| ^1^ Cognitive impairment was defined as scoring in the lowest decile (10%) of each cognitive test. Crude model: No covariates adjusted. Model 1: Adjusted for age, gender, and race. Model 2: Additionally adjusted for education level, smoking status, alcohol consumption, caffeine intake, BMI category, hypertension, diabetes, and energy intake. NPS: Naples Prognostic Score; OR: Odds Ratio; CI: Confidence Interval. | | | | | | |

| Supplementary Table S4. Exploratory stratified analysis of association between NPS and cognitive impairment^1^ | | | |
| --- | --- | --- | --- |
|  | OR (95% CI) | *P* value | *P* for interaction |
| CERAD | | | |
| Age, years |  |  | 0.543 |
| 60–75 | 1.30 (0.99–1.72) | 0.086 |  |
| > 75 | 1.17 (0.75–1.81) | 0.501 |  |
| Gender |  |  | 0.193 |
| Male | 0.96 (0.67–1.36) | 0.815 |  |
| Female | 1.25 (0.83–1.90) | 0.307 |  |
| CFDAST | | | |
| Age, years |  |  | 0.872 |
| 60–75 | 1.51 (1.03–2.23) | 0.059 |  |
| > 75 | 1.56 (1.04–2.34) | 0.054 |  |
| Gender |  |  | 0.109 |
| Male | 2.03 (1.38–2.97) | 0.004 |  |
| Female | 1.27 (0.95–1.70) | 0.127 |  |
| CFDDS | | | |
| Age, years |  |  | 0.614 |
| 60–75 | 1.63 (1.08–2.47) | 0.040 |  |
| > 75 | 1.59 (1.05–2.41) | 0.049 |  |
| Gender |  |  | 0.595 |
| Male | 1.53 (0.85–2.74) | 0.179 |  |
| Female | 1.62 (1.06–2.46) | 0.045 |  |
| ^1^ All models were adjusted for age, gender, race, education level, smoking status, alcohol consumption, caffeine intake, BMI category, hypertension, diabetes, and energy intake. NPS: Naples Prognostic Score; CERAD: the Consortium to Establish a Registry for Alzheimer's Disease; CFDAST: Animal Fluency test; CFDDS: Digit Symbol Substitution Test; OR: odds ratio; CI: confidence interval. | | | |
